# First Brazilian expert consensus for video capsule endoscopy and balloon-assisted enteroscopy^☆^

**DOI:** 10.1016/j.clinsp.2026.100980

**Published:** 2026-05-08

**Authors:** Adriana Vaz Safatle-Ribeiro, Camila Andrade Marinho Farias Diniz, Cesar Amorim, Ellen Francioni Lima Teixeira, Fernando Henrique Porto, Paula Bechara Poletti, Sandrone Granja de Abreu, Ana Botler, Márcio Roberto Facanali, Vitor Arantes, Herbert Toledo

**Affiliations:** Sociedade Brasileira de Endoscopia Digestiva (SOBED), Brazil

**Keywords:** Video capsule endoscopy, Balloon-assisted enteroscopy, Small bowel, Consensus, Therapeutic endoscopy

## Abstract

Video capsule endoscopy and balloon-assisted enteroscopy have changed the management of small bowel lesions. Both methods allow for the examination of the entire small bowel, and balloon-assisted enteroscopy enables therapeutic interventions. Due to the heterogeneous distribution of medical resources across Brazilian states, small bowel endoscopic procedures, though well established in developed countries, have been implemented in a different fashion in Brazil.

**Objective:**

This study aimed to present the results of a Brazilian consensus voting about the major indications for small bowel endoscopy for diagnostic and therapeutic procedures.

**Methods:**

A group of ten members of the small bowel endoscopy committee from the Brazilian Society of Digestive Endoscopy elaborated statements about pre, intra, and post-procedure considerations, diagnostic and therapeutic indications, and complications. An expert panel of 46 endoscopists specialized in small bowel endoscopy across the country revised and discussed each statement and voted utilizing the Delphi methodology. If a minimum threshold of 80% agreement was achieved, the statement was included in the document as a recommendation.

**Results:**

A total of 45 statements were elaborated by the expert panel. All statements were appreciated by the specialists and approved with an 80% (or greater) agreement using the Delphi consensus.

**Conclusions:**

An expert group approved the First Brazilian consensus in small bowel endoscopy utilizing the Delphi methodology, producing a list of 45 statements and recommendations about diagnostic and therapeutic procedures. This consensus may assist the management and decision-making process in patients with small bowel diseases.

## Highlights

• VCE and BAE have changed the management of small bowel lesions.
• VCE is a noninvasive method allowing examination of the entire small bowel.
• BAE enables therapeutic interventions.
• This is the first Brazilian consensus addressing SBE.
• This consensus may assist the medical team in the decision-making process.

## Introduction

It has been almost 25-years since the inception of Video Capsule Endoscopy (VCE) and Balloon-Assisted Enteroscopy (BAE) in medical practice. Both methods have been incorporated into the algorithms of small bowel disease management. VCE represents a noninvasive diagnostic method allowing examination of the entire small bowel, which may serve as a road map for a more invasive procedure such as BAE. BAE includes single-balloon or double-balloon methods. With the combination of antegrade and retrograde routes, BAE allows the examination of the entire small bowel in about 70% of cases.^1^, ^2^, ^3^, ^4^

VCE represents the method of choice for patients with suspected mid-Gastrointestinal (mid-GI) bleeding without signs of obstruction. BAE is the preferred method for patients with signs of obstruction and for patients with altered anatomy, and has been mostly used to perform therapeutic procedures, avoiding surgical interventions in many cases.

Despite having prestigious international consensus and guidelines on Small Bowel Endoscopy (SBE), there is no Brazilian consensus on this topic. Different strategies on the timing and use of VCE and BAE according to different resources and expertise are described in this consensus. Additionally, gastrointestinal diseases of distinct etiology, including infectious diseases, may often involve the small bowel in tropical countries. The first Brazilian consensus also provides guidance to the medical team on the management of small bowel diseases in developing countries.

## Methods

Ten endoscopists from the SBE committee of the Brazilian Society of Digestive Endoscopy (SOBED) organized a questionnaire, which was sent by e-mail to all SOBED members to identify endoscopists who perform SBE in Brazil. All the endoscopists who completed the questionnaire and had performed >50 VCE procedures, or >50 BAE procedures, were invited to participate in the consensus. Forty-six endoscopists agreed to participate.

After systematically reviewing the literature on SBE indications, contraindications, and complications (using the terms small bowel endoscopy, capsule endoscopy, enteroscopy, balloon-assisted enteroscopy, balloon-assisted endoscopy), the committee developed the preliminary statements. The literature search was done in PubMed, Embase, Scopus and Cochrane databases up to September 2024, including randomized trials, meta-analysis and systematic reviews, observational studies and case reports. Only studies published in English were considered.

Forty-five statements were grouped into categories: pre, intra and post procedure, diagnostic and therapeutic indications, and complications. Each statement was voted on by the participants. Only participants with experience in VCE could vote on questions evolving VCE; and in the same way, only participants with experience in BAE could vote on questions evolving BAE.

The online meeting was held in October 2024. Using a link sent by SOBED, the small intestine committee and invited expert endoscopists participated in the online platform voting according to the Delphi consensus survey.^5^ The answers included: “strongly disagree”, “disagree”, “neither agree nor disagree”, “agree” and “strongly agree”. Statements were accepted when they reached a consensus level of 80% or higher. Statements reaching <80% consensus were revised, then entered a second round of the Delphi consensus panel voting, or in a third round if additional revisions were necessary. Out of 46 participants, 35 voted on VCE statements, and 24 voted on BAE statements.

### *Classification of the level of evidence and grade of recommendation*

Following the approval of the statements by the Delphi Consensus, the SOBED Small Bowel Committee classified each statement regarding the quality of the available scientific evidence and the strength of the recommendation, based on internationally accepted criteria (adapted from the GRADE system and the Oxford Centre for Evidence-Based Medicine) (Table 1). The classification system employed is as follows:

**Table 1:** Level of evidence and grade of recommendation according to GRADE system and the Oxford Centre for Evidence-Based Medicine.

Level of Evidence (LE)	Description
I (High)	High-quality evidence; Well-conducted Randomized Controlled Trials (RCTs) or meta-analyses of RCTs
II (Moderate)	Moderate-quality evidence; RCTs with limitations, high-quality cohort studies, or meta-analyses of such studies
III (Low)	Low-quality evidence; Case-control studies, case series, or lower-quality cohort studies
IV (Very Low)	Very low-quality evidence; Expert opinion, case reports, or unsystematic studies
**Grade of Recommendation (GR)**	**Clinical Implication**
A (Strong)	Strong recommendation. Benefits clearly outweigh risks. Based on LE I (High)
B (Moderate)	Moderate recommendation. Benefits likely outweigh risks. Based on LE II (Moderate)
C (Weak)	Weak recommendation. Benefits and risks are balanced or uncertain. Based on LE III (Low)
D (Opinion)	Expert opinion. Based on LE IV (Very Low)

## Results

Of 23 statements on VCE, 16 reached consensus in the first round. Seven statements were discussed and revised by the members of the small intestine committee and were approved after a second round of voting. Of 22 statements on BAE, 14 reached consensus in the first round. Eight statements were discussed and revised by the members of the small intestine committee and were approved after a second round of voting. Three statements needed an additional revision and were approved in a third round.

This paper lists the statements in order of conducting the procedures and provides additional support and references for the statements. The paper concludes with recommendations for the use of BAE and VCE for the diagnosis and treatment of several gastrointestinal diseases (Table 2, Table 3).

**Table 2:** Summary of all statements and final percentage agreement reached in the three rounds.

	Statement	Topic	Agreement 1st Round	Agreement 2st Round	Agreement 3st Round
**1**	On the day before VCE, it is recommended that patients consume a low-fiber diet for breakfast and lunch, and only clear liquids in the afternoon to improve the quality of preparation.	VCE	86%	−	−
**2**	Osmotic laxatives may enhance the quality of small bowel visualization, and their use is at the discretion of the medical team and the availability of the product.	VCE	56%	93%	−
**3**	Antifoaming agents are beneficial in preparing for VCE because it improves the small bowel visualization quality and should be used routinely.	VCE	75%	89%	−
**4**	The routine use of prokinetics is not recommended in VCE, as they do not significantly improve completion rates.	VCE	89%	−	−
**5**	Non-Steroidal Anti-Inflammatory Drugs (NSAIDs) should be suspended, if possible, for at least 4 weeks before VCE.	VCE	77%	100%	−
**6**	Orally administered iron-based drugs should be withheld at least 7-days prior to VCE.	VCE	98%		−
**7**	In patients with swallowing disorders, the video capsule should be positioned directly into the duodenum, with the aid of endoscopy. For children under 8-years of age, the ability of swallowing or the need for endoscopic positioning should be evaluated case-by-case.	VCE	94%	−	−
**8**	The use of cardiac pacemakers, Implantable Cardioverter Defibrillators (ICDs) and Left Ventricular Assist Devices (LVADs) do not represent a contraindication to VCE examination.	VCE	78%	97%	−
**9**	The use of VCE during pregnancy is not recommended. The paucity of data from the literature does not allow any specific recommendation, however it's use could be considered in life-threatening situations, such as massive bleeding.	VCE	92%	−	−
**10**	In situations where there is a need to assess the patency of the small intestine prior to VCE, enteroresonance, enterotomography and patency capsule are advised options, and the choice should depend on the context, expertise and availability of the method.	VCE	97%	−	−
**11**	The patient should be monitored with real-time image visualization (real-time viewer) during the first hour of the examination until the video capsule passes into the duodenum.	VCE	71%	80%	−
**12**	It is recommended that the patient remain fasting, with the intake of clear liquids permitted after real-time visualization of video capsule passage into the duodenum using the real-time viewer, and light food intake allowed 4 h after the start of the examination.	VCE	77%	94%	−
**13**	It is recommended that the examination be concluded only after confirming the passage of the video capsule into the colon through real time visualization (real-time viewer), capsule evacuation or depletion of the recorder's battery.	VCE	97%	−	−
**14**	In patients where the video capsule has not reached the cecum during the recording period, and it has not been evacuated after 14-days; an imaging study should be performed to confirm its retention.	VCE	94%	−	−
**15**	The preferred approach for video capsule retrieval should be endoscopic, reserving surgical intervention only in cases of failure or when surgical indication is warranted due to the original pathology.	VCE	100%	−	−
**16**	It is suggested to read the images at an average speed of 10 frames per second, with the screen display mode as a single image (Single View), reducing the speed when evaluating the proximal small intestine. Digital chromoscopy is not routinely recommended throughout the recording reading, as it does not appear to improve the detection or characterization of lesions.	VCE	68%	96%	−
**17**	The indication for BAE should be well-founded to ensure greater diagnostic positivity, thus reflecting on the quality of the procedure.	BAE	100%	−	−
**18**	BAE should be performed in a hospital environment or in clinics with infrastructure for performing advanced anesthetic procedures.	BAE	70%	77%	85%
**19**	It is recommended to carry out a pre-procedure evaluation to know the clinical history (comorbidities, previous surgeries, allergies), to identify high-risk patients and plan the initial route.	BAE	100%	−	−
**20**	For anterograde BAE, it is only necessary to fast for 8‒12 h. For the retrograde route, bowel preparation is required, similarly to colonoscopy.	BAE	92%	−	−
**21**	The choice of BAE route should be made based on previous imaging tests or VCE results.	BAE	92%	−	−
**22**	In cases of non-definition by imaging or endoscopic exams or failure to perform them, the clinical picture should guide the choice of the access route; if melena: oral route, if enterorrhagia: anal route.	BAE	69%	75%	80%
**23**	General anesthesia is recommended for BAE in pediatric patients, the elderly and those with comorbidities, as well as in therapeutic procedures.	BAE	92%	−	−
**24**	Ideally, Carbon Dioxide Insufflation (CO_2_) should be used, especially in cases where there is an intention to use both routes (greater success in deep intubation and complete enteroscopy).	BAE	96%	−	−
**25**	Fluoroscopy as an adjunct to BAE is useful in special situations, such as altered anatomy, dilation of stenosis, ERCP and placement of self-expanding metal stents.	BAE	100%	−	−
**26**	In case of perforation during BAE, closure of the mucosa with metal clips may be initially considered. If this is not possible, the patient should be referred for surgical treatment.	BAE	100%	−	−
**27**	After BAE, medical reevaluation is essential before the patient is discharged, especially in therapeutic cases such as polypectomies, ERCP, hemostatic procedures, and stent placement.	BAE	100%	−	−
**28**	VCE can be used in the diagnostic evaluation of iron deficiency, with or without anemia, after negative clinical, laboratory, radiologic, and endoscopic investigations using upper gastrointestinal endoscopy and colonoscopy.	VCE	94%	−	−
**29**	It is recommended that, in the investigation of overt mid-gastrointestinal bleeding, VCE should be the first-line small bowel endoscopic examination, provided it is available, due to its safety profile and diagnostic accuracy. It should be performed as early as possible, preferably within 48-hours.	VCE	97%	−	−
**30**	Patients with mid-gastrointestinal bleeding, where VCE identifies lesions amenable to endoscopic treatment, should be referred for therapeutic BAE. However, in the absence of VCE, BAE can be performed as the initial diagnostic procedure.	BAE	78%	92%	−
**31**	In cases of overt bleeding, in patients with hemodynamic stability, it is recommended to perform BAE within 72-hours.	BAE	79%	**100%**	−
**32**	In cases of bleeding with a negative VCE and persistence or recurrence of bleeding, it is recommended to repeat this procedure or perform BAE.	BAE	88%	−	−
**33**	In cases of active bleeding in the emergency setting, with normal upper and lower gastrointestinal endoscopy, VCE or even diagnostic and therapeutic BAE may be performed, depending on availability and expertise.	BAE	78%	92%	−
**34**	In the suspicion of active and severe hemorrhage in the small bowel, cross-sectional imaging (angiotomography) is a diagnostic option prior to endoscopic exams.	BAE	75%	**91%**	−
**35**	VCE is indicated in diagnostic and topographic investigations of Crohn's disease of the small bowel, when there is strong clinical suspicion in the absence of conventional endoscopic and radiological findings.	VCE	98%	−	−
**36**	VCE can be used to study the small bowel to evaluate the response to treatment of Crohn's disease.	VCE	89%	−	−
**37**	In patients with obstructive symptoms and signs, or known stenosis, cross- sectional imaging examination should be performed before BAE.	BAE	92%	−	−
**38**	In inflammatory bowel disease, BAE is indicated for diagnostic confirmation, establishment of differential diagnoses and therapy (dilation and removal of foreign bodies).	BAE	96%	−	−
**39**	VCE can be used in the evaluation of the small bowel in patients with refractory celiac disease, in patients with alarm signs such as anemia, bleeding or weight loss, for differential diagnosis with other enteropathies and possible tumors.	VCE	100%	−	−
**40**	VCE can be used to assess the extent of small bowel polyp involvement.	VCE	97%	−	−
**41**	In patients with polyposis, BAE is recommended for polypectomies to avoid complications such as bleeding, obstruction, intussusception, malignancy, in addition to multiple surgical resections with the risk of short bowel.	BAE	100%	−	−
**42**	VCE can be used in cases of strong diagnostic suspicion of small bowel tumors not identified in imaging tests, especially in cases of suspected neuroendocrine tumor and metastatic melanoma.	VCE	97%	−	−
**43**	If a tumor lesion is suspected, BAE is indicated for performing biopsies, assessing tumor location and extent, and for therapeutic intervention such as placement of self-expanding metal stents.	BAE	79%	96%	−
**44**	In patients with diarrhea, protein-losing enteropathy, or small bowel wall thickening, BAE should be indicated to perform biopsies for differential diagnosis of infectious diseases.	BAE	92%	−	−
**45**	In patients with altered anatomy, BAE is the endoscopic method of choice for diagnostic and/or therapeutic evaluation.	BAE	71%	77%	84%

**Table 3:** Summary of all statements, level of evidence and grade of recommendation.

	Statement	Level of evidence	Grade of recommendation
**VCE Pre-procedure**			
1	On the day before VCE, it is recommended that patients consume a low-fiber diet for breakfast and lunch, and only clear liquids in the afternoon to improve the quality of preparation	III (Low)	C
2	Osmotic laxatives may enhance the quality of small bowel visualization, and their use is at the discretion of the medical team and the availability of the product.	I (High)	B
3	Antifoaming agents are beneficial in preparing for VCE because it improves the small bowel visualization quality and should be used routinely.	I (High)	A
4	The routine use of prokinetics is not recommended in VCE, as they do not significantly improve completion rates.	I (High)	A
5	Non-Steroidal Anti-Inflammatory Drugs (NSAIDs) should be suspended, if possible, for at least 4 weeks before VCE.	III (Low)	C
6	Orally administered iron-based drugs should be withheld at least 7-days prior to VCE.	III (Low)	C
**VCE Procedure**			
7	In patients with swallowing disorders, the video capsule should be positioned directly into the duodenum, with the aid of endoscopy. For children under 8-years of age, the ability of swallowing or the need for endoscopic positioning should be evaluated case-by-case.	III (Low)	C
8	The use of cardiac pacemakers, Implantable Cardioverter Defibrillators (ICDs) and Left Ventricular Assist Devices (LVADs) do not represent a contraindication to VCE examination.	II (Moderate)	B
9	The use of VCE during pregnancy is not recommended. The paucity of data from the literature does not allow any specific recommendation, however it's use could be considered in life-threatening situations, such as massive bleeding.	IV (Very low)	D
10	In situations where there is a need to assess the patency of the small intestine prior to VCE, enteroresonance, enterotomography and patency capsule are advised options, and the choice should depend on the context, expertise and availability of the method.	II (Moderate)	B
11	The patient should be monitored with real-time image visualization (real-time viewer) during the first hour of the examination until the video capsule passes into the duodenum.	II (Moderate)	B
12	It is recommended that the patient remain fasting, with the intake of clear liquids permitted after real-time visualization of video capsule passage into the duodenum using the real-time viewer, and light food intake allowed 4-hours after the start of the examination.	IV (Very Low)	D
13	It is recommended that the examination be concluded only after confirming the passage of the video capsule into the colon through real-time visualization (real-time viewer), capsule evacuation or depletion of the recorder's battery	III (Low)	C
**VCE Post-procedure**			
14	In patients where the video capsule has not reached the cecum during the recording period, and it has not been evacuated after 14-days; an imaging study should be performed to confirm its retention.	III (Low)	C
15	The preferred approach for video capsule retrieval should be endoscopic, reserving surgical intervention only in cases of failure or when surgical indication is warranted due to the original pathology.	III (Low)	C
16	It is suggested to read the images at an average speed of 10-frames per second, with the screen display mode as a single image (Single View), reducing the speed when evaluating the proximal small intestine. Digital chromoscopy is not routinely recommended throughout the recording reading, as it does not appear to improve the detection or characterization of lesions.	II (Moderate)	B
**BAE Pre-procedure**			
17	The indication for BAE should be well-founded to ensure greater diagnostic positivity, thus reflecting on the quality of the procedure	III (Low)	C
18	BAE should be performed in a hospital environment or in clinics with an infrastructure for performing advanced anesthetic procedures	III (Low)	C
19	It is recommended to carry out a pre-procedure evaluation to know the clinical history (comorbidities, previous surgeries, allergies), to identify high-risk patients and plan the initial route.	IV (Very Low)	D
20	For anterograde BAE, it is only necessary to fast for 8‒12 h. For the retrograde route, bowel preparation is required, similar to colonoscopy.	IV (Very Low)	D
21	The choice of BAE route should be made based on previous imaging tests or VCE results.	II (Moderate)	B
22	In cases of non-definition by imaging or endoscopic exams or failure to perform them, the clinical picture should guide the choice of the access route; if melena: oral route, if enterorrhagia: anal route.	IV (Very Low)	D
**BAE Procedure**			
23	General anesthesia is recommended for BAE in pediatric patients, the elderly and those with comorbidities, as well as in therapeutic procedures	III (Low)	C
24	Ideally, Carbon Dioxide (CO_2_) insufflation should be used, especially in cases where there is an intention to use both routes (greater success in deep intubation and complete enteroscopy)	I (High)	A
25	Fluoroscopy as an adjunct to BAE is useful in special situations, such as altered anatomy, dilation of stenosis, ERCP and placement of self-expanding metal stents.	III (Low)	C
26	In case of perforation during BAE, closure of the mucosa with metal clips may be initially considered. If this is not possible, the patient should be referred for surgical treatment.	IV (Very Low)	D
**BAE Post-Procedure**			
27	After BAE, medical reevaluation is essential before the patient is discharged, especially in therapeutic cases such as polypectomies, ERCP, hemostatic procedures, and stent placement.	IV (Very Low)	D
**Indications**			
*Mid-GI Bleeding*			
28	VCE can be used in the diagnostic evaluation of iron deficiency, with or without anemia, after negative clinical, laboratory, radiologic, and endoscopic investigations using upper gastrointestinal endoscopy and colonoscopy.	II (Moderate)	B
29	It is recommended that, in the investigation of overt mid-gastrointestinal bleeding, VCE should be the first-line small bowel endoscopic examination, provided it is available, due to its safety profile and diagnostic accuracy. It should be performed as early as possible, preferably within 48 h.	I (High)	A
30	Patients with mid-gastrointestinal bleeding, where VCE identifies lesions amenable to endoscopic treatment, should be referred for therapeutic BAE. However, in the absence of VCE, BAE can be performed as the initial diagnostic procedure.	II (Moderate)	B
31	In cases of overt bleeding, in patients with hemodynamic stability, it is recommended to perform BAE within 72-hours.	III (Low)	C
32	In cases of bleeding with a negative VCE and persistence or recurrence of bleeding, it is recommended to repeat this procedure or perform BAE.	II (Moderate)	B
33	In cases of active bleeding in the emergency setting, with normal upper and lower gastrointestinal endoscopy, VCE or even diagnostic and therapeutic BAE may be performed, depending on availability and expertise.	II (Moderate)	B
34	In the suspicion of active and severe hemorrhage in the small bowel, cross- sectional imaging (angiotomography) is a diagnostic option prior to endoscopic exams.	II (Moderate)	B
*Crohn’s Disease*			
35	VCE is indicated in diagnostic and topographic investigations of Crohn's disease of the small bowel, when there is strong clinical suspicion in the absence of conventional endoscopic and radiological findings.	II (Moderate)	B
36	VCE can be used to study the small bowel to evaluate the response to treatment of Crohn's disease.	II (Moderate)	B
37	In patients with obstructive symptoms and signs or known stenosis, cross- sectional imaging examination should be performed before BAE.	II (Moderate)	B
38	In inflammatory bowel disease, BAE is indicated for diagnostic confirmation, establishment of differential diagnoses and therapy (dilation and removal of foreign bodies).	III (Low)	C
*Celiac Disease*			
39	VCE can be used in the evaluation of the small bowel in patients with refractory celiac disease, in patients with alarm signs such as anemia, bleeding or weight loss, for differential diagnosis with other enteropathies and possible tumors.	II (Moderate)	B
*Polypoid Syndromes*			
40	VCE can be used to assess the extent of small bowel polyp involvement.	III (Moderate)	C
41	In patients with polyposis, BAE is recommended for polypectomies to avoid complications such as bleeding, obstruction, intussusception, malignancy, in addition to multiple surgical resections with the risk of short bowel.	III (Low)	C
*Tumors*			
42	VCE can be used in cases of strong diagnostic suspicion of small bowel tumors not identified in imaging tests, especially in cases of suspected neuroendocrine tumor and metastatic melanoma.	II (Moderate)	B
43	If a tumor lesion is suspected, BAE is indicated for performing biopsies, assessing tumor location and extent, and for therapeutic intervention such as placement of self-expanding metal stents.	III (Low)	C
*Infectious Diseases*			
44	In patients with diarrhea, protein-losing enteropathy, or small bowel wall thickening, BAE should be indicated to perform biopsies for differential diagnosis of infectious diseases.	III (Low)	C
*Altered Anatomy*			
45	In patients with altered anatomy, BAE is the endoscopic method of choice for diagnostic and/or therapeutic evaluation.	III (Low)	C

## Video capsule endoscopy

### *Pre-procedure*

Statement 1 On the day before VCE, it is recommended that patients consume a low-fiber diet for breakfast and lunch, and only clear liquids in the afternoon to improve the quality of preparation.

#### Level of evidence III, grade of recommendation C

Statement 2 Osmotic laxatives may enhance the quality of small bowel visualization, and their use is at the discretion of the medical team and the availability of the product.

#### Level of evidence I, grade of recommendation B

The optimal preparation for VCE remains controversial. As with colonoscopy, the presence of residues and bubbles in the small intestine can interfere with Small Bowel Visualization Quality (SBVQ) and the diagnostic yield.^6^ Initially, the recommendation was to adopt a low-fiber diet on the day before the exam, clear liquids in the afternoon, and fasting 8- to 12-hours before the procedure. In its latest review, the European Society of Gastrointestinal Endoscopy (ESGE) recommends a low-fiber diet and clear liquids as preparation for the exam, despite a low level of evidence.^7^

The use of osmotic laxatives for the preparation for VCE can be used depending on personal preference or institutional practices.^8^ However, evidence shows that the use of cathartic agents may improve SBVQ. There is a lack of national literature supporting the ideal laxative in Brazil and a standard dosage.

Currently, regimens using Polyethyleneglycol (PEG) and electrolytes are considered the first choice for international studies.^9^ Vikram S. et al. in a systematic review, analyzed 15 studies comparing patients who were not prepared for the exam with those who received PEG and concluded that the SBVQ improves significantly with laxative use.^10^ In a meta-analysis including 982 patients, Shan Wu et al., observed superiority in the SBQV using 2-L of PEG compared to those using 1-liter, 4-L, or only fasting. A slight increase in diagnostic yield was noticed, although it did not affect the Completion Rate (CR).^11^ Two other meta-analyses^12^^,^^13^ also demonstrated superiority in SBVQ with the use of PEG in preparation for VCE and showed that the regimen using 2-L solutions showed good tolerability and efficacy.

Chen H. et al. in a prospective randomized clinical trial, randomly divided the patients into 4-groups. Group A consumed a clear liquid diet after lunch on the day before VCE, followed by overnight fasting. Group B took 250 mL 20% mannitol and 1-liter 0.9% saline orally at 05:00 h on the day of the procedure. In group C, the same regimen was taken at 20:00 h on the day before and at 05:00 h on the day of VCE. In group D, in addition to the group C regimen, 20 mL oral simethicone was taken 30-minutes before VCE. Results showed that mannitol used in split doses with the addition of simethicone improved SBVQ when compared with 10 h of overnight fasting alone.^14^ These regimens could be used as an alternative to PEG in Brazil.

Statement 3 Antifoaming agents are beneficial in preparing for VCE because it improves the small bowel visualization quality and should be used routinely.

#### Level of evidence I, grade of recommendation A

Wu L. et al. published a meta-analysis comparing 121 patients who were prepared for the exam with laxatives, clear liquids, and fasting with another group of 121 patients who also received simethicone. Adequate or excellent/good visualization of the small bowel mucosa was achieved in a statistically significant percentage of patients receiving simethicone compared to those without simethicone (66.1% vs. 37.2%). There was no statistical difference in the completion rate.^15^

In another meta-analysis that included 10 studies, Chen S. et al. analyzed primary outcomes related to the reduction of bubbles and the SBQV in groups with and without simethicone. As secondary outcomes, they examined diagnostic yield, gastric transit time, small bowel transit time, and the completion rate. Compared to the control group, the simethicone group showed significant improvement in reducing bubbles (95% CI, *p* < 0.001) and in SBQV (95% CI, *p* < 0.001). No statistical improvement was seen in secondary outcomes.^16^

Several other studies support the use of antifoaming agents in the preparation for VCE examinations, as they significantly improve SBQV by reducing the presence of bubbles, particularly in the distal portions of the small bowel.^17^, ^18^, ^19^

Statement 4 The routine use of prokinetics is not recommended in VCE, as they do not significantly improve completion rates.

#### Level of evidence I, grade of recommendation A

The use of prokinetics does not seem to provide benefits, in normal situations, for the completion rate, and is therefore not recommended for routine VCE. Vikram S. et al. studied the effect of laxatives, antifoaming agents, and prokinetics in preparing for VCE. A group of 188 patients who received prokinetics before VCE was compared with 189 patients who did not. No statistically significant difference was observed in the completion rate between patients who received prokinetics compared to those who did not (86.7% vs. 75%, respectively, OR = 2.02; 95% CI 0.77–5.27, I^2^ = 63).^10^

In patients at increased risk of incomplete VCE or in those in whom the video capsule remains in the stomach for a prolonged time (confirmed by real-time viewer), the use of prokinetics may provide benefits.^7^

In equipment models with 8-hour battery life, about 20% of exams did not reach the cecum due to recording time limitations. With new technologies featuring 12-hour battery life, the effects of prokinetics on the rate of incomplete examinations are minimal.^6^^,^^20^

Statement 5 Non-steroidal anti-inflammatory drugs (NSAIDs) should be suspended, if possible, for at least 4-weeks before VCE.

#### Level of evidence III, grade of recommendation C

NSAIDs account for 5% to 10% of all drug prescriptions in developed countries and account for about 25% of reported side effects, including gastroduodenal ulcers, which are observed in 10% to 30% of patients.^6^

NSAIDs alter intestinal permeability within approximately 12-hours of ingestion and can cause mucosal inflammation in the short term. A variety of endoscopic lesions have been described, ranging from asymptomatic enteropathy to severe lesions such as ulcers, perforation, and stenosis.^6^

In a study by Maiden L et al., 40 healthy volunteers underwent baseline VCE and fecal calprotectin test. After receiving diclofenac 75 mg (with omeprazole 20 mg twice a day for gastric protection) for 14-days, both tests were repeated. After treatment, 30 subjects (75%) had increased fecal calprotectin levels above the normal limit. VCE revealed new changes in 27 subjects (68%). The most common lesions were erosions (16 subjects, 40%), followed by petechiae or erythematous spots (13 subjects, 33%), denuded mucosa (8 subjects, 20%), blood in the lumen without an identified focus (3 subjects, 8%), and bleeding (2 subjects, 5%).^21^

Severe enteropathies, such as circumferential ulcers with strictures, are also described in approximately 2% of patients with long-term use of low doses of acetylsalicylic acid. Short-term use can also cause small bowel lesions, manifesting as multiple petechiae, erythematous spots, decreased villi, erosions, or ulcers. Discontinuing NSAIDs for at least 4-weeks before VCE is recommended, as these drugs can cause mucosal lesions mimicking those seen in Crohn's disease.^22^

Statement 6 Orally administered iron-based drugs should be withheld at least 7-days prior to VCE.

#### Level of evidence III, grade of recommendation C

The diagnostic yield of VCE may be limited by reduced visualization of the mucosal surface due to dark residues, bubbles, or debris, especially in the distal small intestine.

The use of oral iron supplements can form dark enteric residue, hiding the intestinal mucosa and even mimicking melena. This significantly impairs mucosal visibility. For this reason, it is recommended to suspend iron-based oral medications for a period of at least 7-days before the procedure.^23^^,^^24^

### *Procedure*

Statement 7 In patients with swallowing disorders, the video capsule should be positioned directly into the duodenum, with the aid of endoscopy. For children under 8-years of age, the ability to swallow or the need for endoscopic positioning should be evaluated on a case-by-case.

#### Level of evidence III, grade of recommendation C

Patients with swallowing disorders are at greater risk for tracheobronchial aspiration, a rare but troublesome complication of VCE.^7^^,^^25^ For this reason, the authors should routinely question all patients, especially the elderly, about swallowing difficulties.

In known or suspected cases of swallowing disorders (e.g., dysphagia due to stroke sequelae), it is recommended to place the video capsule by endoscopy, directly into the duodenum. Ideally, if available, it is recommended to use the capsule delivery device, since it allows the capsule to be released easily and safely, with adequate propulsion and without mucosal trauma.^26^, ^27^, ^28^, ^29^ If the delivery device is unavailable, other devices can be used in an adapted manner for direct endoscopic positioning, such as snare and roth net, or a specific endoscopic retrieval device, being aware of technical difficulty and risk of local trauma.

In children, if there is no formal contraindication (e.g., oropharyngeal dysfunction, changes in esophageal or gastric motility), children over the age of 8-years should be able to swallow the video capsule conventionally. Children under 8-years of age should be questioned, along with their parents, regarding their ability to swallow the device. For children under 4-years of age or those with psychological or emotional resistance, endoscopic placement should also be considered.^30^^,^^31^ In a meta-analysis with 995 pediatric patients, 88.4% swallowed the video capsule without difficulties, with the youngest patient in this series being 4-years-old.^32^ The use of the video capsule for children over 2 years of age has been approved by the FDA since 2009.^33^

Statement 8 The use of cardiac pacemakers, Implantable Cardioverter-Defibrillators (ICDs), and Left Ventricular Assist Devices (LVADs) does not represent a contraindication to VCE examination.

#### Level of evidence II, grade of recommendation B

Previous recommendations of video capsule manufacturers formally contraindicated their use in patients with implantable cardiac devices, such as pacemakers, ICDs, and LVADs, due to the theoretical risk of interference. However, technical specifications for video capsules that use radiofrequency data transmission demonstrate that the maximum electromagnetic transmission capacity is below the limits permitted for these cardiac devices.

For this reason, *in vivo* and *in vitro* studies were performed to evaluate the actual interference between video capsule and cardiac devices, concluding that patients can safely undergo the procedure when indicated.^34^, ^35^, ^36^, ^37^, ^38^, ^39^ Several observational studies have demonstrated that the video capsule does not interfere with the cardiac pacemaker functioning and that the pacemaker does not interfere with the VCE’s ability to capture images.

Although there are fewer data in the literature evaluating video capsule interference in patients with ICDs and LVADs when compared with pacemakers, all existing evidence reinforces the safety profile of performing the exam. Harris et al. evaluated 108 patients with cardiac devices who underwent VCE, and did not identify any changes in the device functioning or in the previously programmed parameters (comparatively evaluated before and after the exam).^35^ However, brief periods of image lapse on the video capsule film (< 2 min) were reported in patients with LVAD, without compromising its functioning.^35^

In a retrospective multicenter study of patients with different types of pacemakers and ICDs who underwent VCE, no clinically significant event or impairment in the functioning of these devices was reported.^39^ Likewise, an *in vitro* study did not find any interactions between 21 types of pacemakers and VCE, despite the proximity of the two devices.^36^

For these reasons, the consensus recommends that patients with cardiac devices can safely undergo VCE, as they do not present clinically relevant adverse effects or interferences.

Statement 9 The use of VCE during pregnancy is not recommended. The paucity of data from the literature does not allow any specific recommendation; however, its use could be considered in life-threatening situations, such as massive bleeding.

#### Level of evidence IV, grade of recommendation D

Data from the literature on the use of VCE during pregnancy is insufficient to make clear recommendations. Although few, the case reports available resulted in favorable outcomes for both the mother and the fetus.^40^^,^^41^

Due to the scarce literature, the consensus recommended against the use of VCE during pregnancy, although it can be considered in extreme and life-threatening situations, such as massive bleeding. In these cases, the procedure should be performed only after extensive discussion with the patient and family, warning about potential risks and benefits, both for the mother and the fetus. In other situations, whenever possible, the examination should be postponed until the post-pregnancy period.

Statement 10 In situations where there is a need to assess the patency of the small intestine prior to VCE, enteroresonance, enterotomography, and patency capsule are advised options, and the choice should depend on the context, expertise, and availability of the method.

#### Level of evidence II, grade of recommendation B

Video capsule retention represents the most worrisome complication of the procedure. The rate of retention varies depending on the clinical indication, being higher in patients with established Crohn's Disease (CD).^42^^,^^43^ Even so, the retention risk is low in established CD in the absence of obstructive symptoms, known stenosis, or previous small intestine resections. In this scenario, careful clinical history is the best tool to estimate the risk and assess whether additional complementary exams are necessary.

Therefore, the indiscriminate use of complementary exams to prevent video capsule retention is not necessary in every patient who will undergo the exam.^42^

Cross-sectional imaging (CT/MRI enterography) or a patency capsule is recommended when there is clinical suspicion of obstruction (abdominal pain, abdominal distension, nausea, vomiting, constipation) or identification of a relevant predisposing factor for stenosis.^44^, ^45^, ^46^

Some other conditions identified in the patient's clinical history increase the risk of compromised intestinal patency, thus requiring additional study prior to performing VCE, including previous enterectomies, pelvic/abdominal radiotherapy, and chronic use of high-dose NSAIDs.^44^^,^^47^, ^48^, ^49^ In these cases, CT/MRI enterography or patency capsule is advised, and the choice should depend on the availability and expertise with the method.

Studies comparing patency capsule and cross-sectional imaging methods in patients at high risk for retention are limited, and the results are conflicting. Yadav et al. showed substantial equivalence between the use of the patency capsule and cross-sectional imaging,^50^ while a multicenter Italian study showed significantly lower retention rates (0.7%) in high-risk patients with a negative patency capsule than in those with negative results on cross-sectional imaging (8.3%).^51^

Cross-sectional imaging studies dedicated to the small bowel may overestimate or have low specificity and low positive predictive value in the assessment of stenosis.^50^, ^51^, ^52^ Therefore, the use of the patency capsule may be recommended even in cases of negative findings on cross-sectional modalities in patients with suspected CD and obstructive symptoms. In 2016, a study reinforced this theory, demonstrating capsule retention in high-risk patients with negative findings on CT enterography.^51^ Rosendorn et al. evaluated the ability of MRI enterography to predict retention. Due to the low specificity (59%) and low PPV (40%) in predicting retention, the authors also recommended the use of a patency capsule before a conventional capsule in at-risk patients, regardless of MRI enterography findings.^52^

Unfortunately, the patency capsule is not widely available in some regions of Brazil, due to its cost and lack of coverage by health insurance companies.

Statement 11 The patient should be monitored with real-time image visualization (real-time viewer) during the first hour of the examination until the video capsule passes into the duodenum.

#### Level of evidence II, grade of recommendation B

One of the main causes of incomplete small bowel examination is prolonged gastric transit time. Literature indicates that the capsule transit time in healthy volunteers takes up to 4 hours. However, patients with a history of abdominal surgery, delayed gastric emptying, diabetic neuropathy, severe hypothyroidism, chronic renal failure, use of psychotropic drugs or narcotics, reduced mobility, and patients undergoing the examination while hospitalized may have significantly prolonged transit times, leading to incomplete small bowel evaluation.^20^^,^^53^

Previous studies have defined capsule retention in the stomach for >1- or 2-hours after ingestion as a failure of gastric-to-small bowel passage.^54^

Prokinetics have been used to aid capsule passage from the stomach to the small bowel. However, the isolated use of prokinetics has proven ineffective in increasing completion rates of capsule endoscopy.^20^ Nevertheless, in patients with risk factors for prolonged gastric transit time, certain prokinetics (metoclopramide, domperidone, or erythromycin) may be beneficial when the video capsule remains in the stomach for >30- to 60-minutes, as confirmed by real-time monitoring. ESGE recommends the use of prokinetics and/or endoscopy-assisted capsule administration into the duodenum for patients with confirmed delayed gastric emptying via real-time monitoring.^53^

A prospective study^55^ conducted in Japan compared small bowel VCE in 80 patients with and without real-time visualization. In this study, if the video capsule remained in the stomach for >60-minutes in the real-time visualization group, a prokinetic was administered, followed by PEG. The completion rate in the real-time visualization group was significantly higher than in the control group (90% vs. 72.5%). Similarly, a recent study demonstrated that the use of a real-time viewer increased small bowel completion rates from 66% to 86% (*p* = 0.002).^56^

In a study by Tontini et al., real-time monitoring demonstrated a positive impact and cost-effectiveness in patients at higher pretest risk for incomplete examinations, including those with conditions suggesting gastroparesis, those with a history of incomplete VCE, and patients undergoing the examination while hospitalized.^54^

Statement 12 It is recommended that the patient remain fasting, with the intake of clear liquids permitted after real-time visualization of video capsule passage into the duodenum using the real-time viewer, and light food intake allowed 4-hours after the start of the examination.

#### Level of evidence IV, grade of recommendation D

When VCE was first introduced into clinical practice, manufacturers recommended that patients maintain absolute fasting during the first 2-hours after capsule ingestion. After this period, water intake was permitted, and after 4-hours, a light meal was allowed. In the absence of studies evaluating the effect of the timing of water and/or food intake on capsule image quality and/or transit time, current European and American guidelines, endorse adherence to this regimen, based on expert opinion but without supporting evidence of adequate quality.^7^^,^^29^

However, recent experience, primarily derived from studies focused on preparation regimens for colon capsule endoscopy, suggests that earlier ingestion of clear liquids may improve both capsule image quality and propulsion.^7^^,^^29^ In the systematic review by Nandhra et al., gastric transit time of video capsules in healthy individuals ranged from 0.4- to 1-hour when fasting, and from 2.4- to 3.5-hours when administered with a diet. Small bowel transit time ranged from 3.3- to 5.7-hours, corroborating the notion that allowing the intake of clear, translucent liquids after confirming capsule passage into the small intestine may be an interesting strategy, not only regarding image quality but also for video capsule progression through the small bowel.^57^ Until further studies are complete, the consensus recommends following the recommendation of the manufacturer.

Statement 13 It is recommended that the examination be concluded only after confirming the passage of the video capsule into the colon through real-time visualization (real-time viewer), capsule evacuation or depletion of the recorder's battery.

#### Level of evidence III, grade of recommendation C

Small bowel examination via VCE is only considered complete when the capsule reaches the cecum, thus allowing a full endoscopic study of the small intestine. To ensure this objective, in the absence of lesions or abnormalities that might prevent capsule progression, the recommended practice is to continue the examination until depletion of the recorder’s battery. However, in patients where video capsule evacuation is observed during the examination period and/or confirmation that it has reached the colon by real-time viewer visualization, the examination can be concluded.

There is no specific data in the literature supporting that the examination should only be concluded at battery depletion. In cases of urgent exam reading, such as active bleeding, concluding the examination after evaluating the small intestine may expedite results by a few hours.

In general, the complete assessment of the small intestine via VCE ranges from 82% to 97% in different case series, with lower rates (77%) in hospitalized patients. In patients undergoing examination for CD or suspected small bowel neoplasia, completion rates vary from 78.2% to 85.4% and 84.2% to 92.2%, respectively. In these patients, in the absence of objective evidence of video capsule entry into the colon, it is suggested to conclude the examination only at battery depletion.^7^^,^^45^^,^^54^^,^^58^

### *Post-procedure*

Statement 14 In patients where the video capsule has not reached the cecum during the recording period, and it has not been evacuated after 14-days, an imaging study should be performed to confirm its retention.

#### Level of evidence III, grade of recommendation C

Video capsule retention is defined as the persistence of the capsule in the small intestine for >14-days.^29^^,^^54^ The capsule retention rate varies according to different indications for examination.

In a meta-analysis by Gerson et al., capsule retention rate was 2.1% in patients with suspected small bowel bleeding (95% CI 1.5%–2.8%), 2.2% (95% CI 0.9%–5.0%) in those undergoing evaluation for abdominal pain and/or diarrhea, 3.6% (95% CI 1.7%–8.6%) in patients under investigation for Inflammatory Bowel Disease (IBD), and 8.2% (95% CI 6.0%–11.0%) in patients with established IBD.^43^

Despite the increasing number of examinations, video capsule retention rates have declined over the years, likely due to improved methodology and better patient selection.^59^, ^60^, ^61^

Statement 15 The preferred approach for video capsule retrieval should be endoscopic, reserving surgical intervention only in cases of failure or when surgical indication is warranted due to the original pathology.

#### Level of evidence III, grade of recommendation C

Video capsule retention is usually asymptomatic and may remain in the small intestine without symptoms for several months or years, being naturally expelled during subsequent follow-up.^7^^,^^29^^,^^60^

However, both BAE and surgery are possible alternatives for capsule retrieval. If there is no surgical indication related to the original pathology, retrieval via BAE represents the best alternative, with success rates ranging from 90% to almost 100%.^7^^,^^29^^,^^60^^,^^62^

Except in cases where surgical intervention is indicated, such as tumors, a conservative approach of observation and patient follow-up should be implemented.^7^^,^^29^^,^^61^

In patients with IBD, the initiation or optimization of medical treatment may promote capsule evacuation in up to 20% to 30% of cases.^7^

Most cases of spontaneous video capsule excretion typically occur within the first 4- to 12-weeks after ingestion, but capsule retrieval can reasonably be considered after 3- to 6-months.^63^

Statement 16 It is suggested to read the images at an average speed of 10-frames per second, with the screen display mode as a single image (Single View), reducing the speed when evaluating the proximal small intestine. Digital chromoscopy is not routinely recommended throughout the recording and reading, as it does not appear to improve the detection or characterization of lesions.

#### Level of evidence II, grade of recommendation B

Structured reading of VCE recordings at an average speed of approximately 10-frames per second (fps) in single-frame viewing mode (Single View) is consistent with quality performance measures defined by the European Society of Gastrointestinal Endoscopy (ESGE), which recommend a maximum of 10 fps in Single View ‒ or up to 20 fps when using Multiview display ‒ with further deceleration during the evaluation of the proximal small bowel to minimize the risk of missed lesions.^64^

Comparative studies have demonstrated that increasing reading speed or using multiframe display modes may shorten review time but are associated with a small, measurable decline in lesion detection.^65^

Therefore, most experts advocate a conservative approach, prioritizing diagnostic accuracy, maintaining a reading speed of 10 fps in Single View, and reducing the speed when evaluating the proximal segment.^66^

Routine use of digital chromoendoscopy (e.g., FICE, Blue Mode) throughout the entire VCE recording is not recommended, since current evidence does not demonstrate consistent improvement in lesion detection or characterization. ESGE explicitly discourages its systematic application,^64^ and a meta-analysis showed no significant gain in overall diagnostic yield compared with standard white-light reading.^67^

More recent reviews suggest that virtual chromoendoscopy may enhance visibility of specific vascular or pigmented lesions ‒ such as angioectasias ‒ but its benefit remains situational rather than universal.^68^

## Balloon-assisted enteroscopy (BAE)

### *Pre-procedure*

Statement 17 The indication for BAE should be well-founded to ensure greater diagnostic positivity, thus reflecting on the quality of the procedure.

#### Level of evidence III, grade of recommendation C

The diagnostic yield in BAE seems to be associated with two main factors: patient selection and its use after an abnormal finding in VCE or an imaging dedicated to the small intestine.^69^ However, there are some variations in the diagnosis yield between different centers (53.3% to 81.2%). These variations likely reflect the differences in the various indications for BAE and the different levels of experience among endoscopists. In the study of the English group (UK Quality Improvement Project), adherence to the list of ESGE guided indications (bleeding to be clarified, iron deficiency anemia, evaluations of small bowel tumors, IBD, polyposis, etc.) for BAE was associated with significantly higher diagnostic yields compared to procedures performed for other nonspecific indications.^69^

Statement 18 BAE should be performed in a hospital environment or in clinics with an infrastructure for performing advanced anesthetic procedures.

#### Level of evidence III, grade of recommendation C

The invasive nature of BAE, combined with the long procedure time, requires high doses of sedation to ensure comfort and quality of the procedure. A study of 956 patients undergoing different endoscopic modalities under conscious sedation showed that the tolerability of BAE was worse than that of other endoscopic modalities, including Endoscopic Retrograde Cholangiopancreatography (ERCP), despite high doses of sedation. Therefore, almost all BAE procedures require deep sedation with propofol or general anesthesia to be performed comfortably.^70^

Due to the increased risks from general anesthesia or deep sedation compared to conscious sedation, BAE should always be performed in places with advanced anesthetic support.^69^

Statement 19 It is recommended to carry out a pre-procedure evaluation to know the clinical history (comorbidities, previous surgeries, allergies), to identify high-risk patients and plan the initial route.

#### Level of evidence IV, grade of recommendation D

As with any endoscopic examination, careful acquisition and review of the patient's history is essential for planning an adequate BAE, predicting difficulties, determining sedation strategies and the choice of the best initial route.^69^

Statement 20 For anterograde BAE, it is only necessary to fast for 8‒12 hours. For the retrograde route, bowel preparation is required, similar to colonoscopy.

#### Level of evidence IV, grade of recommendation D

Anterograde BAE follows the same fasting aspects as upper digestive endoscopy. Thus, fasting for 8- to 12-hours is necessary for adequate visualization of the mucosa. For the retrograde route, it is necessary that the patient undergo bowel preparation.^7^

Statement 21 The choice of the BAE route should be made based on previous imaging tests or VCE results.

#### Level of evidence II, grade of recommendation B

BAE might be preceded by less invasive methods, such as imaging of the small bowel (CT or MRI enterography) or VCE.^7^ These methods may approximate the location of a small bowel lesion and guide the insertion of the BAE route either orally or anally. Gay et al.^71^ conducted a study in which an index based on the transit time of the capsule along the small intestine was used to decide the BAE route. In this study, retrograde BAE was indicated if any lesion was found after 75% of the estimated transit time by VCE. The positive and negative predictive values of this index based on VCE transit time were 94.7% and 96.7%, respectively, predicting with certainty the best route for the BAE to be performed. Only about 12% of the cases required a second BAE by the alternative route.

Statement 22 In cases of non-definition by imaging or endoscopic exams or failure to perform them, the clinical picture should guide the choice of the access route; if melena: oral route, if enterorrhagia: anal route.

#### Level of evidence IV, grade of recommendation D

Although VCE and imaging tests of the small intestine are very helpful in guiding the BAE route, these methods might be expensive or not fully available everywhere. Additionally, the exams may not be fully informative.

In these scenarios, clinical presentation should guide the decision-making process. In the presence of melena (probably indicating lesions in the mid and upper digestive tract), the anterograde route should be chosen, and in the presence of enterorrhagia (suggestive of more distal bleeding), the retrograde route should be chosen.^7^

### *Procedure*

Statement 23 General anesthesia is recommended for BAE in pediatric patients, the elderly and those with comorbidities, as well as in therapeutic procedures.

#### Level of evidence III, grade of recommendation C

The safety of endoscopic sedation is an important issue in gastrointestinal endoscopy. BAE can be performed safely under conscious sedation using drugs such as midazolam, fentanyl and propofol.^72^

However, elderly patients, children, or patients who require therapeutic procedures are considered higher risk for sedation-related complications due to frequent comorbidities, the complexity and long procedure time.^73^ In this group of patients, general anesthesia is recommended.

Statement 24 Ideally, Carbon Dioxide (CO_2_) insufflation should be used, especially in cases where there is an intention to use both routes (greater success in deep intubation and complete enteroscopy).

#### Level of evidence I, grade of recommendation A

BAE is a long procedure that requires gas insufflation into the small intestine for visualization. The use of air insufflation, which is poorly absorbed, impairs the technique due to the air being introduced into the intestinal loops, and causes pain and distention. CO_2_ is better absorbed. In a randomized, controlled, double-blind study using DBE,^74^ CO_2_ insufflation, when compared with air, significantly improved insertion depth and reduced patient discomfort. A similar finding was reported in a controlled, double-blind study using SBE.^75^

In a multicenter study, CO_2_ insufflation with SBE versus air significantly improved insertion depth in patients who had previous surgery, and all patients reported less pain.^76^

Statement 25 Fluoroscopy as an adjunct to BAE is useful in special situations, such as altered anatomy, dilation of stenosis, ERCP and placement of self-expanding metal stents.

#### Level of evidence III, grade of recommendation C

In large retrospective case series, fluoroscopy was mostly applied on demand, depending on the endoscopist's experience and the anatomy of the patient's small intestine.^77^ It is particularly useful at the beginning of the learning curve and in the presence of adhesions in patients with previous surgeries. For patients with long and excluded limbs after bariatric surgery or after gastric resection and gastroduodenopancreatectomy, fluoroscopy may help to guide the direction. In addition, it is recommended in therapeutic cases such as small bowel dilation and stent placement.^7^

### *Contraindications*

The contraindications are similar to those of conventional endoscopy and colonoscopy, especially when there is a risk of perforation due to the friability of the intestinal wall.^1^ In case of latex allergy, enteroscopy with a silicone overtube is available in both single-balloon and double-balloon platforms.^78^

Statement 26 In case of perforation during BAE, closure of the mucosa with metal clips may be initially considered. If this is not possible, the patient should be referred for surgical treatment.

#### Level of evidence IV, grade of recommendation D

Regarding the adverse events associated with BAE, the most feared is intestinal perforation.

In a systematic review, Xin et al. collected data from 9047 cases and reported that 0.2% (20 patients) were cases of perforation.^79^ Depending on the location and size of the perforation and availability of resources, endoscopic therapy may be an option (such as closure with clips); however, surgery may be required.

### *Post-procedure*

#### Desinfection

Cleaning and disinfection of the device must follow the same current standards determined by the National Health Surveillance Agency – ANVISA used for endoscopes and colonoscopes, remembering the additional channels in the case of the BAE.

#### Discharge of patients from the endoscopy unit

Statement 27 After BAE, medical reevaluation is essential before the patient is discharged, especially in therapeutic cases such as polypectomies, ERCP, hemostatic procedures, and stent placement.

#### Level of evidence IV, grade of recommendation D

Post-procedure clinical evaluation, by both the anesthesiologist and the endoscopist, is a fundamental step. Pain, nausea and abdominal distension must be treated. Patients undergoing short procedures without therapy may be discharged on the same day after accepting a light diet.^80^^,^^81^ However, patients undergoing long-duration procedures, involving bile duct intervention or the resection of multiple bowel polyps, must remain in the small hospital overnight.^82^^,^^83^ Therefore, medical decisions must be made on a case-by-case basis.

### *Report*

The report should be complete, describing all the findings and treatments performed. Many patients may require additional BAE sessions, such as in cases of multiple vascular lesions or multiple polyps, or even in those who require more than one dilation session. In any case, regardless of the need for a new BAE approach, the description and endoscopic images should be carefully included.

Adverse events occurring during the examination should be noted in the discharge report, which will help minimize the risks and recurrence of the same errors. Databases with relevant information should be created.^53^^,^^84^

Indications for small bowel endoscopy

## ⁠Mid-gastrointestinal bleeding

Statement 28 VCE can be used in the diagnostic evaluation of iron deficiency, with or without anemia, after negative clinical, laboratory, radiologic, and endoscopic investigations using upper gastrointestinal endoscopy and colonoscopy.

### Level of evidence II, grade of recommendation B

Mid-Gastrointestinal (mid-GI) bleeding occurs at any site between the ampulla of Vater and the ileocecal valve. Suspicion arises primarily when no signs of bleeding are detected by upper gastrointestinal endoscopy or colonoscopy.^85^ It may present as either overt or occult bleeding, with the term “obscure gastrointestinal bleeding” reserved for cases in which, even after a complete evaluation of the small intestine, the bleeding source remains unidentified.

In the clinical context of iron deficiency with an unclear etiology, a comprehensive medical history, along with upper gastrointestinal endoscopy including gastric and duodenal biopsies, and ileocolonoscopy, is essential before evaluating the small intestine. If the small bowel evaluation remains necessary to determine the etiology, VCE should be the first-line endoscopic examination due to its broad diagnostic yield and favorable safety profile.^53^^,^^86^, ^87^, ^88^

There is insufficient evidence regarding the role of fecal occult blood testing as a screening tool to determine whether VCE should be performed in cases of unexplained iron deficiency.^53^

Scientific controversy exists regarding the contribution of second-look endoscopy before performing VCE.^89^, ^90^, ^91^ Whilst the American Society for Gastrointestinal Endoscopy (ASGE) recommends repeating upper endoscopy and colonoscopy prior to proceeding with VCE in cases of suspected small bowel bleeding when the initial procedures are nondiagnostic or suboptimal,^92^ the ESGE guideline recommends that the decision to repeat conventional endoscopic procedures should be made on a case-by-case basis, considering the quality of prior investigations and clinical context.^53^ If VCE does not identify a bleeding site, it provides adequate evidence of low risk for rebleeding, with high negative predictive value.

Statement 29 It is recommended that, in the investigation of overt mid-gastrointestinal bleeding, VCE should be the first-line small bowel endoscopic examination, provided it is available, due to its safety profile and diagnostic accuracy. It should be performed as early as possible, preferably within 48-hours.

### Level of evidence I, grade of recommendation A

In various high-quality evidence studies and internationally recognized guidelines,^53^^,^^93^^,^^94^ VCE is recommended as the first-line examination for investigating mid-GI bleeding. Prospective studies have demonstrated its significant superiority, as shown by Leusse A et al.^95^ VCE provides a broader diagnostic yield, a higher complete examination rate, less invasiveness, and greater safety. Despite its limitation on therapeutic interventions, VCE significantly contributes to the therapeutic planning based on the bleeding source and status, guiding the anatomical route for intervention, either surgically or endoscopically by BAE. However, in settings with limited resources, BAE could be performed initially for both diagnosis and therapeutic approach.

According to Wang Z et al., VCE has a higher rate of successful diagnosis compared to CT evaluation.^96^ The diagnostic return of VCE was significantly higher when compared to small bowel radiographic imaging (27% vs. 4%; 95% CI 5%–42%) and angiography (53% vs. 20%; 95% CI 9%–53%; *p* = 0.016).^96^

Leung et al., in a randomized controlled trial, supported the use of VCE over angiography in patients with overt mid-GI bleeding.^97^ Subsequent randomized trial demonstrated significantly greater diagnostic efficacy with VCE compared to push enteroscopy (72.5% vs. 48.7%; *p* = 0.03).^98^

A meta-analysis reported a diagnostic rate of 62% (95% CI 47.3%–76.1%) for VCE and 56% (95% CI 48.9%–62.1%) for BAE, with an odds ratio of 1.39 (95% CI: 0.88–2.20; *p* = 0.16) favoring VCE in patients with mid-GI bleeding.^99^ The performance of BAE was significantly higher when performed after a positive VCE than after a negative VCE.

The timing of its performance influences VCE’s diagnostic accuracy in mid-GI bleeding. VCE performed within 48-hours of the bleeding event increases not only the diagnostic but also the therapeutic yield while reducing hospitalization time.^100^

A propensity score-matching study by Zhao et al., 2021,^101^ involving 997 patients, demonstrated that early VCE had a higher diagnostic rate than late VCE (56.4% vs. 45.5%, *p* = 0.001).

A meta-analysis conducted by Elli L et al., evaluating VCE at 24-, 48-, and 72-hours after the bleeding event, reported diagnostic rates of 83.4%, 81.3%, and 63.6%, and therapeutic intervention rates of 57.6%, 59.1%, and 18.9%, respectively.^102^

Performing BAE within the first 72-hours of the bleeding event is more commonly dependent on VCE being conducted within 48-hours.

Statement 30 Patients with mid-gastrointestinal bleeding, where VCE identifies lesions amenable to endoscopic treatment, should be referred for therapeutic BAE. However, in the absence of VCE, BAE can be performed as the initial diagnostic procedure.

### Level of evidence II, grade of recommendation B

It is widely agreed that VCE and BAE are complementary technologies in the management of mid-GI bleeding. Three meta-analyses have demonstrated similar bleeding findings between the two techniques when used as first-line investigations.^99^^,^^103^^,^^104^

Nakamura et al., 2006, and Kameda et al., 2008, conducted prospective studies in which endoscopists were blinded to VCE findings. These studies confirmed comparable diagnostic rates between VCE and BAE, with no statistically significant differences, though the total number of patients was small (n = 73).^105^^,^^106^

The use of VCE as the first-line approach in mid-GI bleeding also increases the predictive value of BAE, improving the bleeding site identification rate from 56% to 75% in systematic evaluations.^99^ However, this strategy can be adjusted depending on the availability of local resources, equipment, and expertise. The cost-effectiveness economic analyses have suggested that initial investigation with BAE may be a more cost-efficient strategy compared to other diagnostic modalities.^99^

Therapeutic BAE is indicated in approximately 50% to 60% of cases following VCE, for procedures including biopsies, tattooing, polypectomies, hemostasis, as well as endoscopic mucosal resection, dilation of strictures, stent placement, and foreign body removal.^104^

Statement 31 In cases of overt bleeding, in patients with hemodynamic stability, it is recommended to perform BAE within 72-hours.

### Level of evidence III, grade of recommendation C

Although the role of upper and lower gastrointestinal endoscopies in the context of gastrointestinal bleeding is well established, there is limited data on the use of VCE and BAE in the emergency setting for the investigation of mid-GI bleeding. Overt bleeding is defined as the externalization of blood, such as melena or hematochezia. Mönkemüller et al., addressing emergency BAE performed within 24-hours after overt bleeding, suggested that this procedure is feasible and safe with high diagnostic rates, improving patient management and therapy. The presence of visible blood has significant clinical implications, with an increase in BAE positive results.^107^

Another study by an Italian group identified the bleeding site in 92.3% of cases with externalized blood, in contrast to a 12.9% success rate in cases of occult bleeding.^108^ The European Consensus strongly recommends performing a VCE examination as soon as possible, ideally within 48‒72 h after bleeding externalization.^53^

Chao et al. studied the ideal time for performing VCE. They observed detection rates between 70%‒77.6% within the first three days after initial bleeding. However, when VCE was performed on the fourth day, the bleeding site detection rate dropped to 36.4%.^109^ Furthermore, Estevinho et al. demonstrated, in a meta-analysis, lower recurrent bleeding with early VCE and BAE (OR = 0.40; *p* < 0.01).^110^ Considering the Brazilian context and the heterogeneity of available resources, this consensus recommended the ideal time as up to 72-hours to maximize both diagnostic and therapeutic probabilities, whether through VCE or BAE.

Statement 32 In cases of bleeding with a negative VCE and persistence or recurrence of bleeding, it is recommended to repeat this procedure or perform BAE.

### Level of evidence II, grade of recommendation B

The diagnostic rate using VCE is influenced by multiple factors, including the preparation and the interval between bleeding and performance of SBE, findings of hemoglobin <10 g/dL, chronic bleeding (longer than 6-months), more than one episode of bleeding, and exteriorization of bleeding (60% vs. 46% if occult). Lepileur et al. showed that the finding of a visible bleeding lesion indicated a high VCE positive predictive value.^111^ Brito et al., in a meta-analysis, demonstrated that BAE after VCE improves the diagnostic yield.^112^ Interestingly, in patients with negative VCE, BAE diagnosed the bleeding site in about one-third of these patients. It is important to note that there is a lack of evidence regarding the long-term clinical outcome of patients who had negative BAE. However, in patients who were continually investigated due to persistent or recurrent bleeding, about 73.6% of patients had the bleeding site identified using BAE, with 68.8% of the cases located at the mid small bowel.^113^ In this meta-analysis, it was suggested that patients with negative BAE have a low risk of recurrence of bleeding.

Statement 33 In cases of active bleeding in the emergency setting, with normal upper and lower gastrointestinal endoscopy, VCE or even diagnostic and therapeutic BAE may be performed, depending on availability and expertise.

### Level of evidence II, grade of recommendation B

As previously described, VCE and BAE presented comparable diagnostic rates for identifying the bleeding site, thus justifying the use of the available tool.^108^ It suggests that overt suspected mid-GI bleeding is associated with positive findings. This is supported by findings from other investigators. ESGE recommends considering BAE and/or dedicated small-bowel cross-sectional imaging as the first diagnostic tests for patients with suspected mid-GI bleeding, depending on availability, expertise, and clinical suspicion, when VCE is unavailable or contraindicated.^53^

Statement 34 In the suspicion of active and severe hemorrhage in the small bowel, cross-sectional imaging (angiotomography) is a diagnostic option prior to endoscopic exams.

### Level of evidence II, grade of recommendation B

Abdominal angiotomography (CT angiography) captures sectional images in the unenhanced, arterial, and late venous portal phases, enabling angiographic study with multiplanar reconstructions at a low cost, minimally invasive, and with relatively broad availability and speed in the studied country. It allows the diagnosis of the bleeding site with high accuracy (sensitivity of 82.5%‒89% and specificity of 85%‒95%), particularly in the jejunum and ileum. An animal model study demonstrated that CT angiography can detect bleeding as low as 0.3 mL/min.^114^ This technology allows detailed visualization of the small intestine wall and is useful for assessing structural lesions indicated by abnormal wall enhancement, thickening, and contrast extravasation into the lumen. Special attention should be given to patients with acute or chronic renal failure, where the use of venous contrast is not recommended. Patients under 40-years of age and those with active bleeding are associated with higher rates of bleeding detection site.^114^

When associated with bleeding and obstructive signs or symptoms, CT angiography should precede endoscopic exams to guide the best sequential diagnostic approach.^53^ In cases of active bleeding, BAE can be used sequentially after cross-sectional imaging, prioritizing the possibility of therapeutic intervention. If hemodynamic instability, arteriography with embolization should be considered.^114^

## Crohn’s disease

Statement 35 VCE is indicated in diagnostic and topographic investigations of Crohn's disease of the small bowel, when there is a strong clinical suspicion in the absence of conventional endoscopic and radiological findings.

### Level of evidence II, grade of recommendation B

Statement 36 VCE can be used to study the small bowel to evaluate the response to treatment of Crohn's disease.

### Level of evidence II, grade of recommendation B

Statement 37 In patients with obstructive symptoms and signs, or known stenosis, cross-sectional imaging examination should be performed before BAE.

### Level of evidence II, grade of recommendation B

Statement 38 In inflammatory bowel disease (IBD), BAE is indicated for diagnostic confirmation, establishment of differential diagnoses, and therapy (dilation and removal of foreign bodies).

### Level of evidence III, grade of recommendation C

Crohn's Disease (CD) is a chronic inflammatory bowel condition that predominantly affects the terminal ileum, with approximately 80% of cases exhibiting small bowel involvement, and 30% having lesions restricted to the small intestine. Inflammation in this location, especially in the proximal small bowel, represents a diagnostic and therapeutic challenge, often leading to delay in diagnosis and increased risk of complications such as strictures and the need for surgical resections.^115^^,^^116^

Both VCE and BAE offer new perspectives for the management of CD.^116^

VCE, with its high sensitivity (91%‒100%) and specificity (91%‒92%) compared with ileocolonoscopy, is indicated for investigating the small intestine in the absence of obstructive symptoms or known strictures, and in cases of unclassified IBD. Its ability to detect additional lesions in the proximal small bowel, even when colonoscopy reveals only ileocolonic findings, demonstrates its value in the comprehensive assessment of the disease.^98^^,^^117^ In a multicenter, retrospective study conducted by Monteiro et al., which evaluated cases of “unclassified” disease, VCE confirmed the diagnosis of CD in 25%.^118^

The European Crohn's Colitis Organization (ECCO) guidelines establish that BAE can also be used as a diagnostic tool for endoscopic and histological confirmation in cases of negative diagnosis by endoscopy and suspicion by VCE or MRI enterography.^115^ The need to exclude differential diagnoses such as lymphoma, tuberculosis, tumors, lesions caused by anti-inflammatory drugs, among others, is a priority, as treatment with immunosuppressants can worsen the evolution of certain pathologies.^115^

It is relevant to emphasize the importance of a complete medical history and collection of biomarkers of inflammation, since CD doesn’t have a unique pattern or source of diagnosis, but is based on a combination of clinical, biochemical, radiological, endoscopic and histological features.^115^, ^119^ This comprehensive assessment can justify the need for VCE and increase the accuracy of this tool toward active CD of the small intestine findings.

In 21 patients with CD, the sensitivity and specificity for diagnosis of CD of the terminal ileum were 100% and 91% by CE, 81% and 86% by MRI enterography, and 76% and 85% by CT enterography, respectively. ^120^ For patients with clinical and laboratory suspicion of CD, but inconclusive by imaging and conventional endoscopic tests, VCE showed jejunal and ileal lesions in 25/41 cases and CT enterography in 12/41, confirming the disease.^121^

In a pediatric population, a prospective study comparing the accuracy of MRE enterography versus Intestinal Ultrasound (IUS) and VCE to measure inflammatory activity showed no differences between the methods. Aloi et al. did not find significant differences in the accuracy of these three imaging modalities.^122^

VCE should be considered as complementary to colonoscopy in doubtful cases, in order to confirm the diagnosis, and to evaluate the extension of the lesions and its activity index, even if related colonic lesions are detected by colonoscopy.^53^ In some cases, VCE is supported by a treatment-to-target strategy, which recommends a survey of clinical, biochemical and endoscopic findings to confirm disease remission with the established treatment.^53^ Niv et al. highlight in their publication that VCE is a significant predictor of long-term endoscopic remission.^119^ So, based on these findings in literature, this group of experts recommends the use of VCE for monitoring treatment response.

Another interesting fact described by Tai et al. is that VCE was able to change the therapeutic management in 64.6% of cases in their cohort. Also, the presence of proximal findings re-established the score of inflammatory severity of the disease in 19.7% of the exams.^123^

Studies from Jensen MD et al. and Voderholzer WA et al. confirmed a higher detection rate at proximal small bowel with VCE than using imaging techniques such as CT and MRI enterography.^121^, ^124^ Furthermore, VCE is a significant predictor of long-term endoscopic remission, assisting in the monitoring of therapeutic efficacy in the treat-to-target strategy.^121^ It is worth mentioning that the symptomatic response to treatment should not necessarily have a consistent correlation with mucosal healing in Crohn's disease.^98^^,^^119^

BAE allows direct visualization of the small bowel mucosa, enabling the collection of biopsies for diagnostic confirmation and the exclusion of differential diagnoses, such as lymphoma, tuberculosis, and tumors. The exclusion of these diagnoses is necessary, since treatment with immunosuppressants, commonly used in CD, can worsen the evolution of other pathologies.^125^, ^126^

Despite the advances provided by VCE and BAE, some challenges remain. The presence of strictures or intestinal obstructions is more frequent with isolated small bowel involvement. In cases with suspected CD, a history of radiotherapy, abdominal surgery or with obstructive symptoms, the use of the patency capsule prior to VCE should be done to evaluate the intestinal patency. If obstructive symptoms are confirmed, radiological exams (MRI, CT) should be used instead of VCE in the jejuno-ileal assessment of the disease.^53^

When considering BAE as a therapeutic procedure, it is important to highlight previous multiple small bowel resection/stenoplasty, resulting in strictures and short bowel syndrome. BAE allows deep small bowel intubation and therapeutic procedures, including minimally invasive endoscopic dilation and removal of a foreign body (retained endoscopic capsule or food bezoar). BAE is useful for cases of acute intestinal obstruction and may avoid more invasive surgical procedures.^53^^,^^127^^,^^128^ Over the past 25-years of experience in BAE, the frequency of therapeutic interventions using BAE increased significantly.^129^ For this reason, this group of experts recommends BAE as an alternative to surgery to dilate the strictures and to remove a foreign body.

## Celiac disease

Statement 39 VCE can be used in the evaluation of the small bowel in patients with Refractory Celiac Disease, in patients with alarm signs such as anemia, bleeding, or weight loss, for differential diagnosis with other enteropathies and possible tumors.

### Level of evidence II, grade of recommendation B

Refractory Celiac Disease (RCD) is characterized by malabsorption and villous atrophy of a persistent nature, despite the patient properly following a gluten-free diet. RCD is classified into type 1 (RCD-1) and type 2 (RCD-2). The differential diagnosis between the two modalities essentially consists of the presence of aberrant T-cells in the duodenal mucosa in RCD-2, detected by flow cytometry.^130^^,^^131^ RCD-2, although less common, is an essentially pre-malignant condition and has a more severe prognosis, with a mortality rate up to 50% in 5-years.^53^^,^^132^ Patients with RCD-1 have an apparently much lower risk of malignant transformation.^130^^,^^131^ VCE can also contribute by assessing the extent and severity of atrophy,^130^ which is generally greater in patients with RCD-2 compared to RCD-1, with a possible correlation with mortality.^131^^,^^133^ Patients who present this modality of apparently unresponsive celiac disease require responsible and careful diagnostic investigation, in order to identify the potential presence of complications, including RCD, ulcerative jejunoileitis, small intestine adenocarcinoma, and Enteropathy-Associated T-cell Lymphoma (EATL). Such suspicions should be raised in the context of rigorous exclusion of accidental gluten intake in the different scenarios of the patient's dietary history.^53^^,^^131^^,^^134^

Due to such risks, RCD and its unresponsive forms require appropriate monitoring of the small intestine for eventual early detection of neoplastic complications. Recent studies have shown that VCE points to a diagnostic yield close to 50%, followed by BAE for biopsies and confirmation of lesions..^53^^,^^135^, ^136^, ^137^

Thus, pursuing adequate diagnostics for defining cases of unresponsive celiac disease or RCD, the following should be carried out: initial upper digestive endoscopy, to perform duodenal biopsies with appropriate endoscopic technique and anatomopathological study. Then, VCE should be conducted to evaluate the extent and intensity of RCD, in addition to locating the lesions that can be approached through BAE.^53^^,^^138^

## Polypoid syndromes

In patients with polyposis, such as Peutz-Jeghers syndrome and Familial Adenomatous Polyposis, surveillance for polyps by small bowel endoscopy is imperative.

Statement 40 VCE can be used to assess the extent of small bowel polyp involvement.

### Level of evidence III, grade of recommendation C

Statement 41 In patients with polyposis, BAE is recommended for polypectomies to avoid complications such as bleeding, obstruction, intussusception, malignancy, in addition to multiple surgical resections with the risk of short bowel.

*Level of evidence III, grade of recommendation C*

### Familial adenomatous polyposis

Familial adenomatous polyposis (FAP) is an autosomal dominant colorectal cancer syndrome resulting from a germline mutation of the Adenomatous Polyposis Coli (APC) gene on chromosome 5q21. In addition to colorectal cancer, patients have a risk of developing adenocarcinoma of the duodenum and ampulla of Vater that is up to 300-times greater than the general population.^139^^,^^140^ The overall risk of high-grade dysplasia or duodenal carcinoma in patients with FAP at age 50 is approximately 15.2%.^139^ This data highlights the need for ongoing endoscopic surveillance, in addition to prevention and treatment.^141^, ^142^, ^143^, ^144^, ^145^, ^146^, ^147^

ESGE recommends endoscopic surveillance of the proximal small intestine using frontal and lateral viewing endoscopes.^148^ An association has been demonstrated between the intensity of duodenal polyposis and the presence of more distal lesions.^149^^,^^150^ Jejunal and ileal polyps can be found in 40% to 70% of patients with FAP, and VCE is indicated if suspected.^53^

There is little evidence regarding the use of BAE in patients with FAP.^151^, ^152^, ^153^ Some authors report that, if the polyps identified by VCE or imaging techniques are larger than 1 cm, BAE should be used for biopsies or endoscopic resection.^148^^,^^154^ However, in patients with FAP and or altered anatomy with Roux-en-Y reconstruction after gastrectomy or Whipple procedure, BAE is indicated for diagnosis and treatment of the excluded small bowel segment.^155^

VCE in patients with FAP has demonstrated the presence of jejunal polyps in up to 87% of cases.^147^^,^^151^^,^^154^ However, when compared to BAE, VCE was shown to underestimate the size of the lesions, in addition to the impossibility of histopathological confirmation.^152^^,^^153^^,^^156^

The prevalence of jejunal polyposis in patients with advanced duodenal adenomatosis by BAE was 83%, mostly tubular adenomas with low-grade dysplasia located in the proximal jejunum.^157^ This data is comparable with the 90% prevalence of jejunal polyps reported in similar studies using BAE.^151^^,^^153^ However, there is no consensus on the surveillance of this part of the gastrointestinal tract in patients with FAP, or on the preferred diagnostic modality in this setting.

It must be emphasized that the risk of developing adenocarcinoma in the small intestine is not restricted to the duodenum. Thus, cases of jejunal adenocarcinoma in patients who did not undergo prior BAE evaluation have been described in the literature. Ruys et al. described three patients with FAP aged between 57 and 71 who presented advanced duodenal disease and jejunal adenocarcinoma; two of them with unfavorable prognosis.^158^ Adenomas in the jejunum have not been found in patients without duodenal adenomas. Most cases of jejunal adenocarcinoma reported in the literature were described in Spigelman IV patients.^149^^,^^150^^,^^158^

Because patients with Spigelman III and IV duodenal polyposis are potentially surgical, BAE may be individually indicated in this subgroup of patients to assess the extent of jejunal involvement.^148^ BAE should be indicated if therapy is needed. Adenomatous lesions smaller than 5 mm can be resected with biopsy forceps, but in larger lesions with central depression, mucosectomy should be performed. In addition, lesions larger than 2 cm might be resected through Endoscopic Submucosal Dissection (ESD).^53^

### Peutz-Jeghers syndrome

Peutz-Jeghers Syndrome (PJS) is characterized by the presence of hamartomatous polyps in the gastrointestinal tract and melanin pigmentation in mucocutaneous regions. Clinical criteria correspond to: 1) Two hamartomatous polyps anywhere in the gastrointestinal tract; 2) One polyp found in a patient with a family history of PJS or with mucocutaneous pigmentation; and 3) In the absence of polyps, diagnosis can be made if there is a family history of PJS and mucocutaneous pigmentation.^159^

PJS is associated with the mutation of the STK11 gene (also known as LKB1), located on chromosome 19, which is responsible for the enzyme serine‑threonine kinase, and under normal conditions has a tumor suppressor effect. Around 50% are new cases with no family history. When hereditary, it is an autosomal dominant disease (1 in 120,000 live births).^160^

In general, polyps are multiple and vary in size. They are more frequently found in the small intestine (> 75%) and less frequently in the stomach and colon.^161^^,^^162^

Hamartomatous polyps present branched bands of smooth muscle covered by hyperplastic glandular mucosa. They are considered benign but are associated with a higher risk of adenocarcinoma in the small intestine. It is not known whether they originate from the polyps or from associated adenomas.

Small bowel screening in patients with Peutz-Jeghers syndrome should be performed in childhood, starting at 8-years of age or earlier if they present symptoms. Noninvasive methods such as VCE and/or MRE are preferred, reserving BAE for polyp resection, thus avoiding complications such as bleeding, intussusception, and intestinal obstruction.^24^ As the patients get older, the focus is on detecting premalignant and malignant lesions.^161^^,^^162^

VCE can guide the BAE route and has the advantage of a complete small intestine examination. However, studies demonstrate a high number of false-negative lesions with CE.^155^^,^^163^^,^^164^

Polyps larger than 15 mm present a higher risk of intussusception and intestinal obstruction and should be removed by BAE.^144^^,^^165^^,^^166^ It is common to find polyps with surface erosion and signs of recent bleeding. In these cases, endoscopic polypectomy is the best therapeutic approach. Endoscopic resection also reduces the number of intestinal resections, preventing short bowel syndrome.^167^

When the polyp is too large for endoscopic resection, intraoperative enteroscopy with conventional equipment may be indicated for polypectomy or for guiding enterectomy.^168^ In complex cases with multiple large polyps and previous intestinal resections, laparoscopic-assisted balloon enteroscopy represents an alternative method for adhesion lysis and polypectomies in a single procedure; even if this procedure may last a few hours.^168^

Therapeutic strategies have been described in patients with multiple polyps: polyps larger than 2 cm should be removed first, followed by those measuring 5 to 10 mm; polyps larger than 3 cm should be sent for histological analysis due to the risk of association with adenocarcinoma, lesions smaller than 2 cm can be treated with a clip leading them to fall out due to ischemia; polyps should be treated in order and by anal route to prevent intussusception; Laparoscopic-assisted balloon enteroscopy should be performed in cases with many adhesions so the surgeon performs the lysis of adhesions and assists advancing the enteroscope.^169^ Injection of saline solution with epinephrine into the polyp base or pedicle can reduce the risk of bleeding and perforation.^170^

More recently, the strategy of ischemic polypectomy by oral route, with application of a clip at the base of the pedicle, appears to have fewer complications. Additionally, it allows the treatment of a greater number of polyps in less time when compared with conventional endoscopic resection.^171^

## Tumors

Statement 42 VCE can be used in cases of strong diagnostic suspicion of small bowel tumors not identified in imaging tests, especially in cases of suspected neuroendocrine tumor and metastatic melanoma.

### Level of evidence II, grade of recommendation B

Statement 43 If a tumor lesion is suspected, BAE is indicated for performing biopsies, assessing tumor location and extent, and for therapeutic intervention such as placement of self-expanding metal stents.

### Level of evidence III, grade of recommendation C

Although the small intestine comprises 70% to 80% of the total length of the gastrointestinal tract, small bowel neoplasms are rare diseases. Only 5% of gastrointestinal neoplasms and only 1% to 2% of malignant tumors of the gastrointestinal tract develop in the small intestine.^172^

The tumors are often diagnosed at an advanced stage, due to nonspecific symptoms, the low degree of suspicion and the difficulty in evaluating the small intestine.

The most frequent clinical presentations are mid-GI bleeding; weight loss; diarrhea, abdominal pain, intestinal obstruction; and CT alterations, such as a thickened intestinal wall. Patients with suspected small bowel tumors, either by radiological examination or by VCE, may undergo BAE for diagnostic and histological confirmation. Endoscopic tattooing may assist the surgeon in locating the lesion for small bowel resection.

Approximately 90% of small intestine malignant tumors are represented by four histological types: neuroendocrine tumor, adenocarcinoma, lymphoma, and GIST (gastrointestinal stromal tumor). The incidence of malignant tumors in the small intestine increases with age, typically between 60- and 70-years of age. About 90% of cases are diagnosed after the age of 40.^173^^,^^174^

For treating intestinal obstruction due to neoplasia, palliative endoscopic management through self-expandable metallic stents represents an alternative method to surgical decompression, allowing for nutritional improvement and better quality of life. The stents can be placed in the jejunum and ileum through BAE. The first description of small bowel stent placement through BAE was made in 2004 by Yamamoto et al.^1^ The authors reported its use in two patients from a series of 123 patients. Using BAE, the diameter of the accessories is no longer a limiting factor. Additionally, the depth of the accessories became shorter, as they are inserted through the 145 cm overtube instead of the 230 cm endoscope.^175^

## Infectious diseases

Statement 44 In patients with diarrhea, protein-losing enteropathy, or small bowel wall thickening, BAE should be indicated to perform biopsies for differential diagnosis of infectious diseases.

### Level of evidence III, grade of recommendation C

Infectious diseases still represent a relevant cause of small bowel involvement in tropical and developing countries. High suspicion of these etiological agents is imperative, especially in patients with diarrhea and malabsorption syndrome.

Infectious small bowel diseases can be caused by bacteria, fungi, protozoa and viruses such as those resulting from tuberculosis, cryptococcosis, leishmaniasis and cytomegalovirus. They can lead to anemia or malabsorption syndrome due to chronic loss of protein in the feces.^176^ Thus, protein-losing enteropathy or thickened small bowel wall on imaging tests may be due to infectious agents. Worm infestations can also be incidentally diagnosed by both VCE and BAE.

Besides VCE, BAE represents an excellent diagnostic method. Although endoscopic findings are often nonspecific, such as edema, enanthema, friability, lymphangiectasia, or lymph extravasation into the lumen, it allows for targeted biopsies with histological confirmation.^177^

## Altered anatomy

Statement 45 In patients with altered anatomy, BAE is the endoscopic method of choice for diagnostic and/or therapeutic evaluation.

### Level of evidence III, grade of recommendation C

BAE allows the excluded limb access of patients with altered anatomy, including gastrectomy, gastroduodenal pancreatectomy, bariatric surgery, and small bowel transplant, among others. It is therefore the endoscopic method of choice in cases of obscure gastrointestinal bleeding or when neoplasia is suspected, allowing not only the diagnosis but also treatment. Furthermore, it allows balloon enteroscopy-assisted ERCP in patients requiring biliary tree or biliodigestive anastomosis dilation, removal of biliary stones, and treatment with stent placement.^178^^,^^179^

In conclusion, BAE has different platforms and several indications, many of them therapeutic. It is a safe procedure, but complications can occur. The knowledge of potential adverse events and risk factors can minimize their occurrence.

Both pre-procedure criteria as well as those during and after the procedure, represent fundamental steps for a standard quality of excellence during the implementation and development of small bowel endoscopy in an endoscopy unit. Awareness and expertise in all steps can determine the procedure's success.

## Conclusions

This is the first Brazilian consensus addressing SBE. The statements were prepared by a group of ten experts and voted on by 46 endoscopists from different Brazilian states, until the Delphi consensus achieved 80% approval. Heterogeneous distribution of medical resources across Brazilian states, and some distinct and frequent indications of SBE in developing countries (infectious diseases) were considered for preparing the consensus. This consensus may be useful in the management of small bowel disorders and may assist the medical team in the decision-making process.

## Data availability

The datasets generated and/or analyzed during the current study are available from the corresponding author upon reasonable request.

## Declaration of competing interest

The authors declare no conflicts of interest.

## Figures and Tables

**Table:** 

1	Afonso Celso da Silva Paredes
2	Alexandre de Souza Carlos
3	Américo de Oliveira Silvério
4	Arthur Adolfo Parada
5	Aureo Paoliello
6	Barbara Cathala Esberard
7	Caio Cesar Wanderley Salem
8	Carlos Saul
9	Cláudio Hashimoto
10	Clóvis Massato Kuwahara
11	Cristiane Kibune Nagasako da Cruz
12	Daniel de Alencar Macedo Dutra
13	Dorival Zito Filho
14	Edgard Rodrigo Pinheiro Cruz de Medeiros
15	Emília Pereira de Magalhães
16	Fabiana Sartore Guimarães
17	Fábio Gomes Teixeira
18	Fernanda Prata Borges Magalhães Martins
19	Fernando Pavinato Marson
20	Flávio Hayato Ejima
21	Flávio Heuta Ivano
22	Francisco das Chagas Gonçalves de Oliveira
23	Guilherme Francisco Gomes
24	Hélcio Pedrosa Brito
25	Isabel Fonseca Santos
26	José Renato Hauck
27	Juliano Coelho Ludvig
28	Laura Cotta Oernellas Halfeld
29	Lucidio Balduino Leitão
30	Luiz Henrique Mazzonetto Mestieri
31	Marcelo Vicente Toledo de Araújo
32	Mauro Gester
33	Mauro José Wagner Moreira Maia
34	Newton Teixeira dos Santos
35	Pablo Rodrigo de Siqueira
36	Roberto Chiumeo do Nascimento
37	Robson Kiyoshi Ishida
38	Thiago Alonso Domingos
39	Thiago Festa Secchi
40	Vitor Monteiro e Silva
